# Prognostic Value of PLR, SIRI, PIV, SII, and NLR in Non-Muscle Invasive Bladder Cancer: Can Inflammatory Factors Influence Pathogenesis and Outcomes?

**DOI:** 10.3390/cancers17132189

**Published:** 2025-06-28

**Authors:** Francesco Pio Bizzarri, Marco Campetella, Pierluigi Russo, Giuseppe Palermo, Seyed Koosha Moosavi, Francesco Rossi, Lorenzo D’Amico, Antonio Cretì, Filippo Gavi, Enrico Panio, Simona Presutti, Fabrizio Bellavia, Mauro Ragonese, Chiara Ciccarese, Roberto Iacovelli, Maria Chiara Sighinolfi, Marco Racioppi, Emilio Sacco, Bernardo Rocco

**Affiliations:** 1 Department of Urology, Fondazione Policlinico Universitario Agostino Gemelli, 00168 Rome, Italy; 2 Department of Urology, Ospedale Isola Tiberina-Gemelli Isola, 00186 Rome, Italy; 3 Department of Life Science, Health, and Health Professions, University Unilink, 00165 Rome, Italy; 4 Department of Oncology, Fondazione Policlinico Universitario Agostino Gemelli, 00168 Rome, Italy

**Keywords:** bladder cancer, inflammatory factors, non-muscle invasive bladder cancer

## Abstract

**Background/Objectives:** Given the increasing interest in the predictive role of inflammation in oncology, we aimed to assess the association between inflammatory factors (IFs) and the histopathological characteristics of bladder cancer (BC). Our objective was to correlate some of these IFs with BC progression and recurrence, identifying possible new diagnostic tools. **Methods**: We retrospectively analyzed 285 patients (79.8% male, 20.4% female; median age 73) who underwent transurethral resection of the bladder (TURB) between January 2016 and January 2022. The preoperative neutrophil-to-lymphocyte ratio (NLR), platelet-to-lymphocyte ratio (PLR), systemic immune-inflammation index (SII), pan-immune-inflammation value (PIV), systemic inflammation response index (SIRI), and standard clinical variables were collected one month before TURB and evaluated as predictors of recurrence and progression. Patients were stratified using the Youden Index and ROC analysis. Cox regression models were applied to identify independent predictors. **Results**: High-grade tumors were present in 74.6% of cases, and 34% were recurrent. Carcinoma in situ was found in 5%. After 72 months, 53% underwent radical cystectomy, and 13.7% died within 5 years. The optimal cutoffs were PLR 139, SIRI 1.12, PIV 248.49, NLR 2, SII 327. Smoking, primary MIBC, age, and lymph node status were significantly associated with recurrence. Elevated PLR correlated with recurrence and T2 progression (*p* = 0.004). Higher SIRI, PIV, and PLR levels were significantly associated with lymphovascular invasion and nodal metastasis (*p* < 0.05). PLR was linked to recurrence in tumors ≥ 3 cm post-BCG (*p* = 0.004); high SIRI predicted recurrence within 48 months (*p* = 0.05). **Conclusions**: High PLR and SIRI levels were associated with recurrence. Our findings support the emerging role of IFs in predicting BC outcomes and suggest their potential inclusion in future prognostic models.

## Simple Summary

Bladder cancer often returns even after treatment, and predicting which patients are at higher risk of recurrence remains a challenge. Inflammation is known to play a role in cancer development, and certain values from routine blood tests may offer helpful clues. In this study, we examined the relationship between several inflammation-related markers found in blood samples and the recurrence or progression of early-stage bladder cancer. We analyzed data from 285 patients treated at our hospital and found that high levels of certain markers, especially those related to the immune and inflammatory response, were associated with a greater risk of recurrence and aggressive disease features. These results suggest that simple, non-invasive blood tests could help doctors better identify patients who need closer monitoring or more intensive treatment. Our findings may support the use of inflammation-based indices in future tools to guide personalized care in bladder cancer patients.

## 1. Introduction

Bladder cancer (BC) is the tenth most commonly diagnosed cancer globally, with approximately 430,000 new cases each year [1,2]. Among genitourinary malignancies, BC is particularly challenging due to its high recurrence rates and variable disease course [3].

The majority of BCs arise from the urothelium, with non-muscle invasive bladder cancer (NMIBC) accounting for around 75% of newly diagnosed cases [4]. While NMIBC generally has a favorable prognosis, it carries a substantial risk of recurrence and progression to muscle-invasive bladder cancer (MIBC) in up to 25% of patients [5]. Current management relies on the transurethral resection of bladder tumors (TURB), often followed by intravesical instillations of chemotherapy or Bacillus Calmette–Guérin (BCG) and regular follow-up cystoscopies [6].

In recent years, systemic IFs derived from routine blood tests have emerged as potential biomarkers in various malignancies, including urothelial cancers [7,8]. IFs such as the neutrophil-to-lymphocyte ratio (NLR), platelet-to-lymphocyte ratio (PLR), systemic immune-inflammation index (SII), systemic inflammation response index (SIRI), and pan-immune-inflammation value (PIV) have shown prognostic value in multiple oncological settings [9,10].

Despite promising evidence in other cancers, the prognostic value of these indices in NMIBC remains unclear [11,12,13]. Moreover, their correlation with histopathological features such as lymphovascular invasion (LVI), carcinoma in situ (CIS), and tumor multifocality has not been thoroughly investigated [14,15,16,17].

The aim of this study is to assess the prognostic role of PLR, NLR, SII, SIRI, and PIV in patients with NMIBC and explore their association with adverse pathological features and clinical outcomes. By identifying reliable and non-invasive biomarkers, we hope to contribute to improved risk stratification and personalized treatment strategies in NMIBC.

Our primary objective was to assess the association between IFs and tumor recurrence. Secondary objectives included the evaluation of their correlation with adverse pathological features (e.g., CIS, LVI, multifocality, tumor size) and recurrence-free survival (RFS).

## 2. Materials and Methods

### 2.1. Study Design and Setting

A single-center retrospective cohort study was conducted at Policlinco Universitario Agostino Gemelli between 2016 and 2022; the study enrolled all patients diagnosed with urothelial BC who underwent TURB, and we evaluated the association between IFs and histological features. The diagnosis was performed in accordance with European guidelines using urinary cytology, cystoscopy, and imaging (ultrasound and/or CT urography) [18]. The study has been approved by the Ethics Committee (ID 2882, approved by the EC on 5 December 2019).

### 2.2. Patients and Variables

We included patients with histologically confirmed NMIBC who underwent TURB. Patients with a history of MIBC or missing IF data were excluded. Patients with a history of chemotherapy, radiation therapy, or other inflammatory or immune disorders were excluded. Basic patient information, including age, sex, smoking status, diabetes mellitus, body mass index, clinical and pathological T stage, surgical technique (bipolar or monopolar), nodal status, LVi, adjuvant therapy (Mitomycin or BCG), and pathological data were collected. Additionally, one month before surgery, blood test results, including white blood cell count, neutrophil count, eosinophil count, monocyte count, lymphocyte count (109/L), and platelet count (109/L), were retrieved from the hospital’s medical record system. Tumor staging and grading were determined based on the European Association of Urology guidelines. NLR was calculated as neutrophil count (×109/L)/lymphocyte count (×109/L), PLR was calculated as platelet count (×109/L)/lymphocyte count (×109/L), SII was calculated as platelet count (×109/L) × neutrophil count (×109/L)/lymphocyte count (×109/L), and SIRI was calculated as neutrophil count (×109/L) × monocyte count (×109/L)/lymphocyte count (×109/L). Patients after BC diagnosis underwent Re-TURB in case of high-grade disease and received a course of bladder instillation (Mitomycine or BCG) afterward, then underwent follow-up in accordance with European guidelines [3].

Of these patients, 267 received endovesical therapy (80 with Mitomycin C and 197 with Bacillus Calmette–Guérin) according to our treatment schedule, which included one cycle of induction (6 weekly instillations) and two cycles of maintenance (6 monthly instillations). As per guidelines, we performed a re-TURB in 58 patients due to the absence of muscle tissue in the pathology specimen or the presence of a large tumor (>3 cm).

### 2.3. Outcomes

The primary outcome was to establish the relationship between IFs and the rate of recurrences. Secondary outcomes were a range of unfavorable pathological characteristics, including LVi, carcinoma in situ presence, prostatic urethra involvement (PUI), recurrence of BC, advanced tumor stage (pTstage), multifocality of tumor, tumor size, and their correlation with IFs. We also defined relapse-free survival (RFS) as the time from (TURB) to the recurrence of cancer.

### 2.4. Statistical Analysis

The study has been reported according to STROBE checklist (Supplementary Materials) [19]. Data analysis was conducted using STATA/SE version 14. The sample size calculation indicates that a total of 123 patients would be sufficient to achieve 80% power with a significance level of 5%. Given that the study includes 285 patients, the sample appears adequately powered to detect a significant association. We determined optimal cutoff points for each biomarker using ROC curve analysis and the Youden index. Continuous variables were summarized using medians and interquartile ranges, while categorical variables were presented as frequencies and percentages. Patient characteristics were compared using the Mann-Whitney U test for continuous variables and the chi-square or Fisher’s exact tests for categorical variables. A *p*-value less than 0.05 was considered statistically significant. We adjusted for potential confounders, including age, gender, tumor grade and size, presence of CIS, smoking habits, LVi, chemo or immune-therapies, and prior recurrence using Cox proportional hazards models. The predictive performance of each model was assessed using the area under the curve (AUC) derived from ROC analysis. We calculated RFS using the Kaplan–Meier method. Log-rank tests were used to compare differences in these survival rates. To assess the association between IFs and RFS, we performed both univariate and multivariate Cox regression analyses.

## 3. Results

### 3.1. Cohort Characteristics

A total of 285 patients who underwent TURB for BC were retrospectively collected at our hospitals. Demographic and histopathological characteristics are summarized in Table 1. Regarding patient clinical characteristics, a statistically significant association in univariate analysis was found between smoking, primary MIBC, age, and lymph node status at cystectomy (*p*-value < 0.05).

### 3.2. Cutoff Determination and ROC Analysis

**Figure 1:**
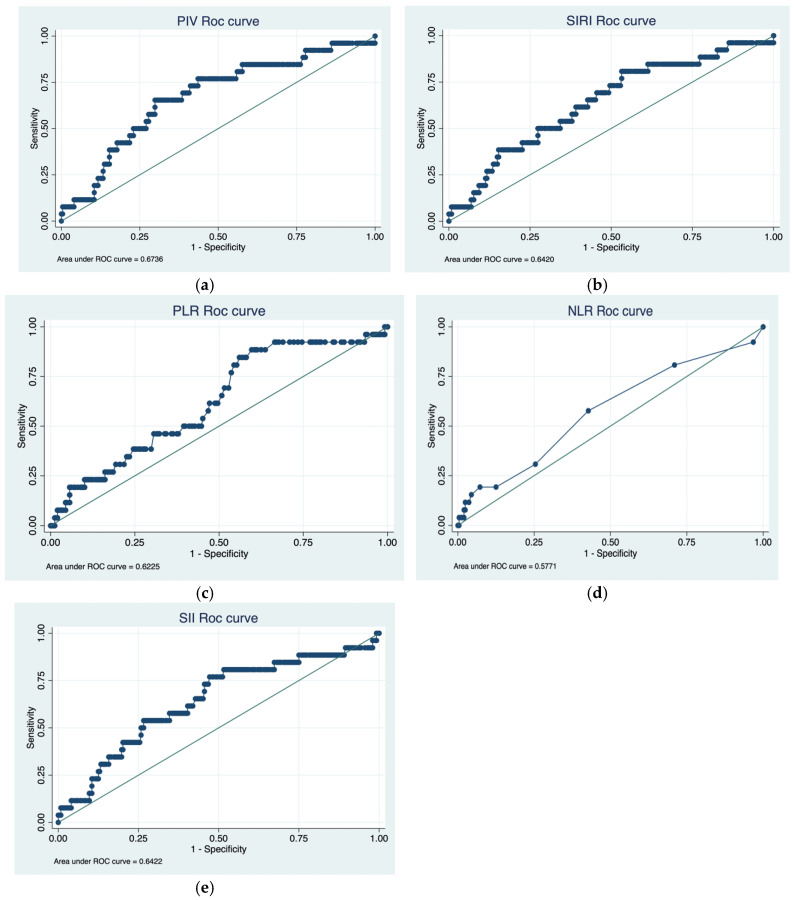
(**a**) ROC curve for prediction of RFS in patients stratified by PIV group, (**b**) ROC curve for prediction of RFS in patients stratified by SIRI group, (**c**) ROC curve for prediction of RFS in patients stratified by PLR group, (**d**) ROC curve for prediction of RFS in patients stratified by NLR group, (**e**) ROC curve for prediction of RFS in patients stratified by SII group.

The median estimates for NLR, SIRI, PIV, PLR, and SII were 3.8 (IQR: 2.1–4.3), 2.42 (IQR: 1.33–5.7), 607 (IQR: 284–1445), 140 (IQR: 124–278), and 1040 (IQR: 520–36,473), respectively. In our multivariate analysis, we found a statistically significant correlation between several histopathological features and IFs (Table 2a,b). Using the Youden index and ROC analysis, we determined the optimal cutoff point for a parameter related to our primary outcomes to be 248.49 for PIV, with an area under the curve (AUC) of 0.67 (Figure 1a). The optimal cutoff for SIRI related to LVi is 1.12, and AUC is 0.63 (Figure 1b); considering PLR, the optimal cutoff is 139 with AUC of 0.59 (Figure 1c). In terms of recurrence, regarding NLR, the optimal cutoff point is 2 with an AUC of 0.57 (Figure 1d); for SII, the cutoff is 327 with an AUC of 0.627 (Figure 1e). We also found a statistically significant association between high levels of SIRI and multifocal disease at univariate and multivariate analysis (*p*-value 0.004).

### 3.3. Oncological Outcomes

**Figure 2:**
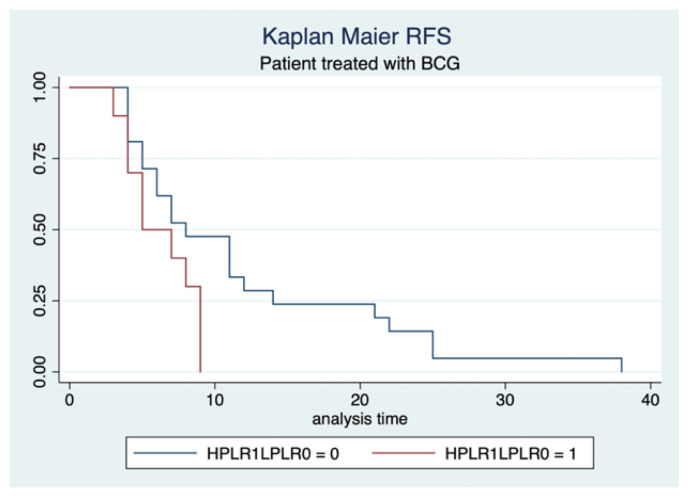
Kaplan–Meier estimates related to RFS.

We used a Cox regression model to analyze the association between IFs and time to recurrence, our primary outcome (Table 2). After adjusting for potential confounders, including smoking status, gender, and clinical T stage, a significant association was observed between elevated PLR levels and recurrence-free survival in patients with tumor size > 3 cm. (HR: 2.5, 95% CI: 2.3–6.8, *p* = 0.04) (Figure 2).

**Figure 3:**
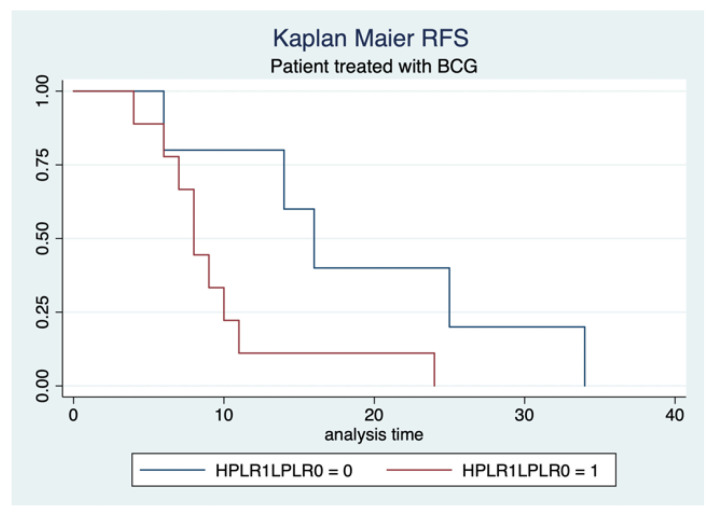
Kaplan–Meier estimates related to RFS.

We found another association between PLR levels and RFS in patients treated with BCG, without differences in terms of tumor dimensions (HR 3.7, 95% CI: 3–14, *p* = 0.04) (Figure 3).

**Figure 4:**
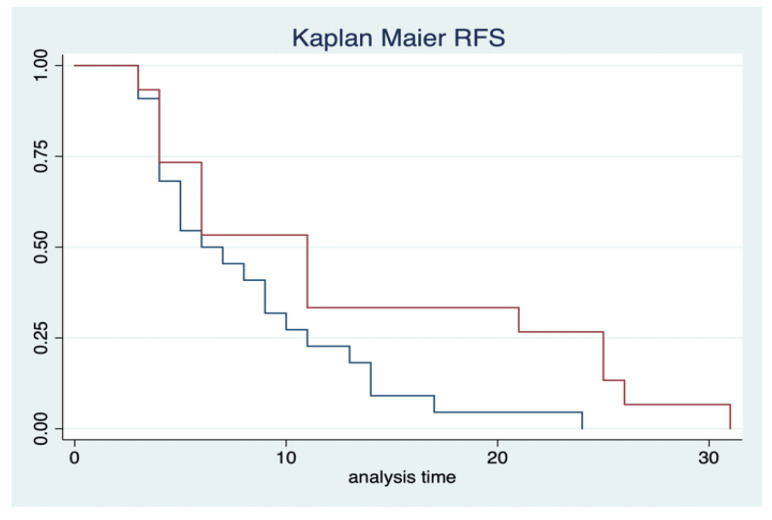
Kaplan–Meier estimates. The red line includes high-level SIRI; the blue one includes low level of SIRI.

Furthermore, we found in terms of RFS, a correlation with a high level of SIRI (HR 0.5; IC 0.4–0.8; *p*-value = 0.05) (Figure 4).

## 4. Discussion

In this study, we investigated the prognostic role of inflammatory markers (IFs) in patients with non-muscle invasive bladder cancer (NMIBC), focusing on their association with recurrence and adverse histopathological features. Our findings confirm that elevated levels of specific IFs—particularly PLR and SIRI—are significantly associated with higher recurrence risk, lymphovascular invasion (LVi), larger tumor size, and aggressive tumor biology.

Chronic inflammation is increasingly recognized as a contributor to tumor initiation, progression, and metastasis [20,21,22]. Inflammation contributes to tumorigenesis by promoting angiogenesis, immune evasion, and genomic instability. Elevated markers like PLR and SIRI may reflect a pro-tumorigenic immune microenvironment favoring recurrence [15]. Our results are consistent with this concept, supporting the hypothesis that systemic IF markers may reflect underlying tumor aggressiveness.

In our cohort, we observed statistically significant associations between lymph node involvement at radical cystectomy and key clinical variables such as smoking status and advanced age. These findings align with previous studies, including Caini et al., which demonstrated that smoking negatively impacts both nodal involvement and overall oncological outcomes in bladder cancer patients [23,24].

The variability in optimal IF cutoffs across studies likely reflects differences in population selection, timing of blood collection, and laboratory methodologies. In our study, the PIV cutoff was set at 248, aligning with values previously reported by Russo et al., who linked high PIV levels to worse outcomes following radical cystectomy [11].

Our SII cutoff (327) differed from Russo’s value of 640 and from Zhang et al.’s threshold of 863, likely due to stricter exclusion criteria in our population (e.g., exclusion of patients with chronic inflammation or prior malignancy) [12]. This underlines the importance of patient selection when evaluating the prognostic utility of IFs.

Our cutoffs for PLR (139) and NLR (2) were similar to those reported by Yilmaz et al. and Chen et al., despite variations in study design. Notably, Yilmaz’s study employed dual blood sampling, both before and after TURB, which may have influenced marker stability [25,26]. In contrast, our single preoperative sampling was deliberately timed to avoid transient inflammatory responses. Our findings are also supported by Prijovic et al., who reported a median SIRI value of 1.53, consistent with our population [27].

Our study demonstrated a statistically significant association between elevated IF levels and recurrence, particularly in patients with large tumors (>3 cm), high-grade cytology, and concomitant carcinoma in situ. These findings are in line with Chen et al., who reported a correlation between higher PLR, NLR, and MLR values and tumor recurrence and size [25].

Furthermore, LVi and nodal metastasis were significantly associated with high IFs. This is supported by studies in both bladder and upper tract urothelial carcinomas, where LVi was identified as an independent predictor of poor outcomes [28]. Salari et al. also demonstrated that elevated PLR, NLR, and SII were predictive of nodal involvement and metastatic progression after radical cystectomy [29].

Lei et al. extended these findings to endometrial cancer, showing that high SII levels correlated with LVi and lymph node metastasis, underscoring the potential of systemic inflammation as a pan-cancer prognostic marker [30].

With regard to progression from NMIBC to muscle-invasive disease (MIBC), our results are consistent with the meta-analysis by Cao et al., which identified elevated SII as a poor prognostic factor for recurrence-free survival (RFS) [31]. In our cohort, higher PLR levels were associated with worse RFS in patients treated with BCG, particularly those with larger, high-grade tumors. This may reflect heightened systemic immune activation in response to aggressive disease. However, it should be noted that while our exclusion of patients with chronic inflammatory conditions reduced confounding, it may also limit the generalizability of our findings.

Our data showed that IFs correlated with key risk variables used in the EORTC risk tables, such as tumor size and presence of CIS. This suggests that inflammation-based indices may enhance existing risk stratification tools and warrants further investigation [32].

Although NLR has been discussed in the European Association of Urology (EAU) guidelines, its prognostic value remains controversial. Wu et al. found a correlation between high NLR values and metastatic disease, with a cutoff close to ours (~2) [16]. However, the SWOG 8710 trial did not confirm NLR as a predictive or prognostic factor, highlighting the need for more robust prospective studies [17].

The strengths of our study include the relatively large sample size, the use of multiple validated inflammatory markers, and robust multivariate modeling. Furthermore, our strict exclusion criteria minimize potential confounding from unrelated inflammatory conditions.

This study has several limitations. First, its retrospective and single-center design may limit external validity. Second, postoperative inflammatory data were not available for all patients. Most importantly, systemic IFs may not fully reflect tumor-associated immune activity, given the heterogeneity of circulating neutrophils, lymphocytes, and monocytes. Future studies should aim to identify tumor-specific immune signatures or circulating immune cell subtypes. Multi-center, prospective studies are essential to validate our findings and evaluate the broader clinical utility of IFs.

### Recommendations and Future Perspectives

The clinical implication of our findings is the potential integration of these easily available blood-based markers into routine risk stratification for NMIBC. In high-risk patients, closer surveillance or early escalation to BCG or radical therapy could be considered.

Our findings suggest that IFs could complement existing tools like EORTC risk tables [33] and align with EORTC risk factors such as tumor size and CIS. Thus, IFs could serve as adjunct biomarkers to refine recurrence risk stratification and tailor surveillance intensity or adjuvant treatment strategies. We recommend validation in prospective multicenter cohorts. Future work should also explore integration with AI-based predictive models and consider longitudinal sampling to track dynamic changes in inflammation [34].

## 5. Conclusions

Systemic IFs such as PLR and SIRI demonstrate prognostic relevance in NMIBC. Their association with recurrence and progression highlights their potential as adjunctive tools for risk stratification and treatment planning. Incorporating these markers into clinical nomograms may enhance individualized patient care and inform future integration of artificial intelligence-driven prognostic models. Further prospective studies are warranted to validate our findings and evaluate the utility of these indices across broader populations and treatment settings. 

## Figures and Tables

**Table 1:** Patients’ demographic characteristics.

Characteristics	Total
Patients	285
Age (mean)	73.2 (34–97)
Gender, n (%)	
Male	226 (79.8%)
Female	67 (20.14%)
Body mass index, median (IQR)	25.74 (23.44–28.08)
Tobacco smoking, n (%)	
Never smoker	54 (19%)
Active smoker	76 (26.86%)
Previous smoker	107 (37.81)
Charlson comorbidity index, n (%)	
CCI < 2	25 (9%)
CCI ≥ 2	259 (91%)
T stage, n (%)	
Tx	45 (15.8%)
Ta	63 (22.18%)
T1	92 (32.39%)
CIS	14 (4.9%)
Concomitant CIS	42 (14.8%)
Prostatic urethra invasion	35 (12%)
Pathological grade 1973	
G1	15 (5.4)
G2	59 (21.2)
G3	190 (68.35)
Pathological grade 2004	
LG	71 (25.62)
HG	209 (74.38)
Size of largest lesion (cm), median	4.4 cm (2.2–6)
Muscle invasion after Re TURB, (n, %)	6 (2%)
Instillation therapy, (n, %)	
Chemotherapy (MMC)	80 (28%)
Immunotherapy (BCG)	197 (69%)
BC recurrences	80 (28%)
HG	64 (80%)
LG	16 (20%)

**Table 2:** Patients’ demographics, disease characteristics, and survival in Cox regression analysis. (a) Statistical analysis: Cox regression model; software: STATA/SE 14; significance level: *p* < 0.05. (b) Statistical analysis: Cox regression model for patients’ characteristics; software: STATA/SE 14; significance level: *p* < 0.05. (c) Statistical analysis: Cox regression model; software: STATA/SE 14; significance level: *p* < 0.05. (d) Statistical analysis: Cox regression model; software: STATA/SE 14; significance level: *p* < 0.05.

**(a)**			
**Characteristics (PLR > 139)**	**Recurrence-Free Survival**		
	HR	95% CI	*p*-value
Age	0.95	0.82–1	0.47
Gender (reference male)			
Female	4.85	0.98–9	0.6
Smoke (reference no)	1.87	1.2–3.5	0.43
LVi	1.6	1.25–5	0.04
Tumor size (>3 cm)	1.4	1.16–3.87	0.05
BCG therapy	0.5	0.32–0.9	0.003
Multifocal disease	1.12	0.7–3	0.9
T stage			
CIS	2.8	1.76–6	0.004
pTa/T1HG	1.2	0.8–2.5	0.89
PUI	0.54	0.36–5.2	0.1
Urinary cytology			
HG	1.7	1.43–4.3	0.03
**(b)**			
**Characteristics (SIRI > 1.12)**	**Recurrence-Free Survival**		
	HR	95% CI	*p*-value
Age	0.73	0.35–1	0.7
Gender (reference male)			
Female	2.46	1.98–6	0.04
Smoke (reference no)	3.12	1.2–7.4	0.002
LVi	0.93	0.33–2.65	0.9
Tumor size (>3 cm)	1.4	1.16–3.87	0.05
BCG Therapy	0.5	0.32–0.9	0.003
Multifocal disease	2.13	0.97–3.9	0.8
T stage			
CIS	0.95	0.55–1.66	0.86
pTa/T1HG	1.16	0.88–1.5	0.7
PUI	3.7	2.12–14	0.02
Urinary cytology			
HG	1.31	0.79–2.18	0.3
**(c)**			
**Characteristics (PIV > 248)**	**Recurrence-Free Survival**		
	HR	95% CI	*p*-value
Age	2.4	0.3–20	0.4
Gender (reference male)			
Female	1.3	0.7–2.4	0.4
Smoke (reference no)	1.1	0.3–3.37	0.9
LVi	2.9	1.5–9	0.02
Tumor size (>3 cm)	1.4	1.16–3.87	0.05
BCG therapy	0.8	0.4–1.8	0.3
Multifocal disease	2.13	0.97–3.9	0.8
T stage			
CIS	2.59	1.5–9.3	0.6
pTa/T1HG	2.7	1.4–4.46	0.001
PUI	4.1	2.3–9	0.2
Urinary cytology			
HG	3.3	2–4.4	0.004
**(d)**			
**Characteristics (SII > 327)**	**Recurrence-Free Survival**		
	HR	95% CI	*p*-value
Age	0.8	0.7–3	0.9
Gender (reference male)			
Female	3.2	1.4–8	0.65
Smoke (reference no)	2.5	1.2–15	0.2
LVi	1.93	0.98–3.5	0.05
Tumor size (>3 cm)	4.3	1.6–9	0.03
BCG therapy	0.9	0.2–3	0.6
Multifocal disease	4.12	1.56–7	0.03
T stage			
CIS	2.14	1.55–12.3	0.6
pTa/T1HG	1.56	0.97–4.76	0.4
PUI	4.5	1.24–12	0.04
Urinary cytology			
HG	2.21	1.18–15	0.5

## Data Availability

The original contributions presented in the study are included in the article, further inquiries can be directed to the corresponding author/s.

## Supplementary Materials

The following supporting information can be downloaded at: https://www.mdpi.com/article/10.3390/cancers17132189/s1.

## Abbreviations

The following abbreviations are used in this manuscript:

NMIBC	Non-muscle invasive bladder cancer
MIBC	Muscle-invasive bladder cancer
NLR	Neutrophil-to-lymphocyte ratio
PLR	Platelet-to-lymphocyte ratio
SII	Systemic immune-inflammation index
SIRI	Systemic inflammation
CIS	Carcinoma in situ
IFs	Inflammatory factors
